# Predictive Factors Associated with Declining Psycho-Oncological Support in Patients with Cancer

**DOI:** 10.3390/curroncol30110707

**Published:** 2023-11-04

**Authors:** Karoline Hecht, Moritz Philipp Günther, Johannes Kirchebner, Anna Götz, Roland von Känel, Jan Ben Schulze, Sebastian Euler

**Affiliations:** 1 Department of Consultation-Liaison-Psychiatry and Psychosomatic Medicine, University Hospital Zurich, University of Zurich, Culmannstrasse 8, 8091 Zürich, Switzerland; moritzphilipp.guenther@usz.ch (M.P.G.); jan.schulze@usz.ch (J.B.S.); sebastian.euler@usz.ch (S.E.); 2 Department of Forensic Psychiatry, University Hospital of Psychiatry Zurich, Lenggstrasse 31, 8032 Zürich, Switzerland; johannes.kirchebner@pukzh.ch; 3 Department of Hemato-Oncology, University Hospital Zurich, University of Zurich, Rämistrasse 100, 8091 Zürich, Switzerland; anna.goetz@usz.ch

**Keywords:** distress in oncological patients, rejecting psycho-oncological support, distress screening, machine learning

## Abstract

(1) Background: International cancer treatment guidelines recommend low-threshold psycho-oncological support based on nurses’ routine distress screening (e.g., via the distress thermometer and problem list). This study aims to explore factors which are associated with declining psycho-oncological support in order to increase nurses’ efficiency in screening patients for psycho-oncological support needs. (2) Methods: Using machine learning, routinely recorded clinical data from 4064 patients was analyzed for predictors of patients declining psycho-oncological support. Cross validation and nested resampling were used to guard against model overfitting. (3) Results: The developed model detects patients who decline psycho-oncological support with a sensitivity of 89% (area under the cure of 79%, accuracy of 68.5%). Overall, older patients, patients with a lower score on the distress thermometer, fewer comorbidities, few physical problems, and those who do not feel sad, afraid, or worried refused psycho-oncological support. (4) Conclusions: Thus, current screening procedures seem worthy to be part of daily nursing routines in oncology, but nurses may need more time and training to rule out misconceptions of patients on psycho-oncological support.

## 1. Introduction

Psychological, social, spiritual, and physical problems summarized under the umbrella term distress are described in 30–50% of patients suffering from cancer [1,2,3]. However, distress often remains undetected in the daily routine and the patients’ need for psychological support often remains unmet [1,4]. Therefore, international cancer treatment guidelines recommend routinely screening cancer patients for distress using screening tools. The intention is that there should be no barriers to psycho-oncological support if distress is present. Usually, patients are asked to self-rate their level of distress and choose the most likely underlying causes from a checklist at regular intervals. Nurses are then required to discuss all screening results with their patients, initiate further evaluations, and offer psycho-oncological support [5]. However, the effectiveness of distress screening tools in obtaining access to psycho-oncology is still under debate [6,7]. Around 35–70% of patients with a positive distress screening decline a psychosocial referral, while on the other hand, 5% of patients without a positive screening demand a referral [8,9,10,11]. Some argue that these shortcomings in screenings are due to a high false positive rate and thus the insufficiency of the instruments used [10,12]. Others argue that this might be a sign that patients are insufficiently informed about the merit of psycho-oncological support. Research on that ongoing discussion found that distressed patients decline help based on their belief that they should be able to manage their emotional situation by themselves (46%) or that their suffering from distress is not severe enough to warrant external help (23%) [13]. (Fear of) stigmatization may also prevent distressed patients from accepting help or even admitting distress [14]. Avoidant coping styles may also lead to the denial of distress as well as psychological needs and are associated with an increased likelihood for major depressive episodes later on [15]. The discrepancy between the screening results and the need for psycho-oncological support highlights the need for a more sophisticated clinical assessment of patients with cancer in addition to routine screening. However, this stands in the way of oftentimes scarce resources [8,9]. Only if we know more details about patients declining psycho-oncological support can we evaluate if screening is insufficient or if we should invest more resources into convincing patients of the benefits of psycho-oncological support. The current study aims to characterize patients who decline psycho-oncological support via patient file data of a large patient population with a machine learning approach, fit to uncover nonlinear hidden correlations. While past research has focused on interviewing distressed patients on their reasons for declining help [13], the present study focuses on a large set of clinically recorded data beyond the level of distress (which may be denied by patients despite its presence–see above). Thus, the present study aims to identify factors predicting a patient with cancer to decline psycho-oncological support.

## 2. Materials and Methods

### 2.1. Measures

At the Comprehensive Cancer Center Zurich (C3Z), Switzerland, where the current study was conducted, institutional guidelines prescribe that every patient diagnosed with cancer should be handed out a distress thermometer (DT) and problem list (PL) at admission. Additionally, patients are asked whether they wish psycho-oncological support. At the C3Z, psycho-oncological support consists of a psycho-oncologist (i.e., clinical psychologist or psychiatrist) discussing the patient’s individual situation in detail and offering tailored help, ranging from social services to psychotherapeutic interventions and psychopharmacology. Screening results are also discussed during nursing reports and recorded in the clinical management software (version 5.0.6, ^®^KISIM byCistec AG, Zürich, Switzerland). At the C3Z, patients with a distress of 5 or higher are screened again after 7 days. Otherwise, screening is repeated after 28 days or in case of readmittance. There are no signs of any patients being preferred over others for screening purposes. The DT is an evidence-based self-rating tool to detect mood disorders in cancer patients [4]. The patient is asked to score his or her distress over the past week on a scale from zero (no distress) to 10 (extreme distress). The PL covers 34 potentially distressing problems arising due to cancer (treatment). The 34 items are categorized into the groups “practical” (e.g., childcare, transport, living), “family” (partner and child), “emotion” (e.g., sadness, fear and depression), “spiritual” (e.g., god), and “physical” (e.g., pain, nausea, sexuality). The patients are asked which problems of this checklist occur in their current situation.

### 2.2. Data Collection

**Figure 1:**
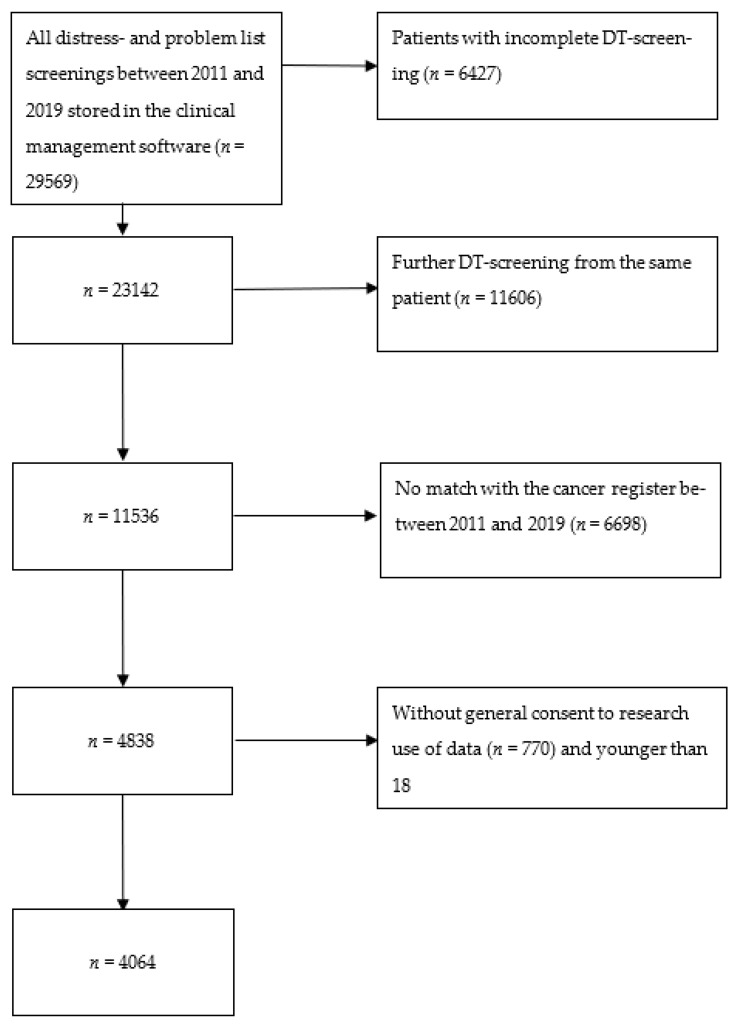
Data collection.

As a first step we collected all DT- and PL-screening results stored in the electronic health record (^®^KISIM, Cistec AG) between 2011 and 2019. Then, we excluded all incomplete screenings. If patients had more than one DT score, we used the first recorded score. In a second step, we matched the DTs with the patient cohort listed in the institutional cancer register (version 5.0, ^®^OncoStar by IT-Choice Software AG, Karlsruhe, Germany) between 2011 and 2019. The register records every patient who is both diagnosed with and treated for cancer at the C3Z, and contains further information (variables) on diagnosis and treatment. Lastly, we also excluded patients younger than 18 years and those who did not provide a general consent to the use of their data for research purposes. Ultimately, the present study included 4064 patients (see Figure 1: Data collection). Almost 800 variables are routinely noted in the electronic health record (^®^KISIM, Cistec AG) and cancer register (^®^OncoStar, IT Choice). Out of these, the authors selected 70 variables with clinical relevance to the research question (for all items see Table A1). All variables were converted to binary variables. To further consider comorbidity, we calculated the Charlson Comorbidity Index (CCI) based on the collected data [16]. Nationality was categorized according to the World Bank classification of countries [17].

Between 2011 and 2019, the proportion of screened individuals to all oncology patients has remained relatively constant around 40%. Similarly, clinical structures remained stable and so did the composition and frequencies of cancers being treated.

### 2.3. Statistical Evaluation

We used machine learning (ML) to identify variables correlating with the declining of psycho-oncological support in order of statistical significance. For the outcome variable (decline psycho-oncological support), the 87% of the 4064 patients declining psycho-oncological support were defined as “No Wish”. Thus, the 13% of patients wishing psycho-oncological support were called “Wish”. Before model building, all cases were divided into a training set (70% of the data) and testing set (30% of the data). To avoid overfitting, we used nested cross-validation (CV) during model building with the training set. We created a 5-fold cross-validation inner loop for the data processing steps and the model training embedded in a 5-fold cross-validation outer loop used for performance testing [18,19]. The testing set was used only to estimate the performance of the final model in the last step.

For all steps described below, R version 3.6.3. and the MLR package v2.171 were used [20]. To estimate the confidence intervals of the balanced accuracy, we used MATLAB R2019a (MATLAB and Statistics Toolbox Release 2012b, The MathWorks, Inc., Natick, MA, USA) with the add-on “computing the posterior balanced accuracy” v1.0.

#### 2.3.1. Inner Loop: Data Processing and Initial Model Building

To be usable for ML, the data needed to be processed in different ways. As a first step, missing values were inferred by the random forest algorithms in the MLR package using the ForestSRC add-on [21]. The reason for this imputation was that some of the ML models only run with a complete data set. Earlier studies have shown that upsampling the minority group seems to be the best way to handle unbalanced data sets in ML. Hence, we upsampled the smaller group of patients who accept psycho-oncological support at the rate of 5.6 in the second step [22]. Exploring the large number of predictor variables, feature selection was conducted to obtain generalizability. Therefore, we pre-selected variables by a random forest algorithm (randomForestSRC package) [18,21]. After finishing the data processing described above, we started building the initial model. First, we tuned the hyperparameters for the different models (for the final hyperparameters, see Table A2). Afterwards, we trained the models with logistic regression, trees, random forest, gradient boosting, k nearest neighbors (KNN), support vector machines (SVM), and Naïve Bayes [23].

#### 2.3.2. Outer Loop: Model Selection

In the outer CV loop of the testing set, every model trained in the inner loop was tested for its balanced accuracy and goodness of fit, sensitivity, specificity, positive predictive value (PPV), and negative predictive value (NPV). Next, we calculated the receiver operating characteristic balanced area under the curve (ROC balanced AUC). The model with the highest AUC was chosen as the best performing model [24].

#### 2.3.3. Final Model Performance

The performance of the final model was verified with the unused testing set. Multicollinearity testing raised no concern for interdependencies within the variables.

## 3. Results

Table 1 shows the characteristics of patients whose data were used in this study.

All of the variables used for the ML are listed in Table A1. Table 2 provides an overview of all of the calculated models and their performance in nested CV (for detailed performance results with confidence intervals, see Table A3).

The best performing model relied on a Naïve Bayes algorithm with a balanced accuracy of 69.2% and an AUC of 79%. The performance measures on the testing set had a balanced accuracy of 68.5% and an AUC of 76%, which is only slightly lower than in the training set during CV. Nevertheless, with a sensitivity of 88.9% and a specificity of 47.2%, the results are relevant (see Table 3: Final Naïve Bayes model performance measures on testing set).

**Figure 2:**
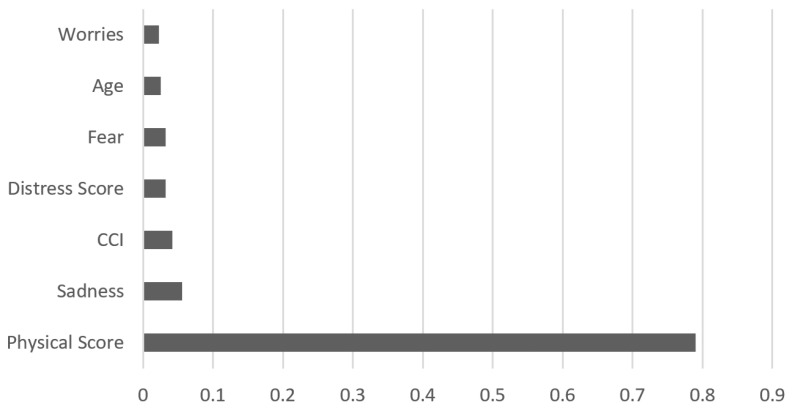
Ranking of most predictive variables for declining psycho-oncological support. Note: This figure indicates the extent (0 to 1.0) to which each of the variables in the final model influences patients’ decisions to accept or decline psycho-oncological support. Physical Score = the total number of physical issues a patient selected on the problem list; CCI = Charlson Comorbidity Index; Fear/Worries/Sadness = Patients who check-marked Fear/Worries/Sadness in the PL.

In summary, our model detects patients who tend to decline psycho-oncological support with a sensitivity of nearly 90%. The most important variables in the final model are age, the CCI, the total number of physical issues a patient selected on the problem list (the “Physical Score”), as well as the items “fear”, “worries”, and “sadness” of the problem list. The latter are all under the category “emotion”. In other words, advanced age, a low CCI, a small total amount of physical items on the problem list, and the absence of “fear”, “worries” and “sadness” lead to a higher probability of declining a referral to psycho-oncology. Figure 2 depicts the weights of the variables in the final model relative to each other. In other words, Figure 2 illustrates to what extent each variable contributes to the decision regarding psycho-oncological support. According to our model, “Physical Score” seems to have the highest impact on declining psycho-oncological support. The absolute and relative distributions of the indicative variables are presented in Table 4.

## 4. Discussion

The aim of this exploratory study was to characterize patients who decline psycho-oncological support and identify factors predicting such refusal. The machine learning based analysis of 4064 patients revealed a model which detects patients who decline a referral to psycho-oncology with a sensitivity of nearly 90%. Overall, 13% of all patients explored in the current study accepted psycho-oncological support. Earlier studies stated similar numbers [9,10]. The acceptance rate tends to be higher in studies in which patients were asked if they request help in general and not specifically psycho-oncological support [12,13].

Our study indicated that older patients, patients with a lower distress score on the DT (meaning little distress), a low CCI, few physical problems, and patients who do not feel sad, afraid, or worried tend to decline psycho-oncological support. Thus, the results support the notion that patients declining psycho-oncological support may do so because they have little distress–or have a denial of distress. Yet, it is remarkable that most of the significant variables are covered with the distress screening procedure, including the PL. This underlines the importance of a thorough screening and justifies the broad application of these instruments to identify psycho-oncological needs. However, there are two variables correlating with declining, which are not covered by the screening tools: age and the CCI.

Remarkably, the total amount of physical burden (physical score) seems to be more relevant in patients’ consideration of psycho-oncological support than the specific symptoms of cancer. Prior studies found the presence of physical symptoms and comorbidities [25,26] as well as “worry” from the problem list to be highly correlated with distress [27]. Thus, it seems consequential that patients with few physical problems and worry on the problem list decline psycho-oncological support. The fact that a low CCI is also indicative of patients declining psycho-oncology seems to provide further support for the validity of physical problems on the problem list being predicative of declining help.

In contrast to physical burden, the specific absence of sadness, fear, and worry (rather than the total number of items) appear to lead patients to refuse help. Remarkably, most PL items correlating with a refusal are form the group “emotions”. One explanation might be that patients assume only fear, sadness, and worries of the problem list to be problems requiring psycho-oncological support. Thus, misconceptions about psycho-oncology (or shame [28]) might misguide patients to refuse support. Alternatively, it is possible that alexithymia (or denial) in some patients may lead to difficulties in the perception of different affective states, which are asked in a quite fine-grained manner by the problem list.

Future research should focus on exploring patients’ understanding of psycho-oncology in order to design targeted information campaigns. Oncological treatment teams should be informed about the specific offers of their psycho-oncological services in order to communicate effectively. For instance, it might be helpful to mention that psycho-oncology includes interventions for effective coping with bodily distress and helps patients to explore their own affective states in order to manage them beyond a global feeling of distress. Ultimately of course, many patients may have sufficient capacity to help themselves or use private networks of social support.

Our study further provides a correlation between higher age and the declining of psycho-oncology. Many studies have shown that not only older patients with cancer, but also the elderly in general, have barriers when it comes to psychological support [9,29,30].

The identified barriers of the elderly are, among others, transportation problems, misconceptions, and stigmatization [31,32]. Addressing patients’ fear of stigmatization, some research proposed to offer psychological support in cooperation with other integrative therapies, which may be easier to accept for some patients [33]. This is in line with Baker-Glenn et al. 2011 and Clover et al. 2015, who propose to offer help more broadly rather than asking specifically for psychological or psycho-oncological needs. In addition, home treatment offers for cancer patients might be helpful to avoid transportation problems. This has been shown in previous studies on patients with depression [29,34].

Additionally, the present study has also identified numerous variables without an association with psycho-oncological needs. These include many sociodemographic parameters such as gender, relationship status, nationality, language, insurance status, occupation, and religion. This is interesting, because in contrast to women, (older) men have been reported to not seek psycho-oncological support [35] or to remain absent from arranged appointments with psychotherapists [36] in prior studies. However, both studies and a review [37] highlight that this phenomenon may wean in younger generations and when efforts are made to improve the fit between men’s psychological needs and the type of psycho-oncological support being offered. Neither cultural nor economic factors seem to influence the subjective need of a patient for psycho-oncological support. Further, a pre-diagnosed mental disorder as well as prescription of psychopharmacology seem to have no influence on the declining or acceptance of a referral. This might indicate that patients with cancer and a mental disorder are not different from patients with just cancer in their self-evaluation for need of psycho-oncology.

The present study has several limitations. First, it cannot differentiate if patients decline psycho-oncology because they feel no authentic need or because they have internal barriers (e.g., fear of stigmatization, misconceptions, alexithymia, denial) to accepting support. However, it is also a strength of the present study that it exclusively relies on routinely documented data in current standardized cancer treatment. While it would be interesting to interview cancer patients on their decision making processes, such research would be hampered in its validity by various psychological phenomena (e.g., patients not being able to reflect thoroughly, patients not wanting to disclose the thoughts or stereotypes that they have, patients wanting to please those asking questions, etc.). The results we provide are relevant (and clinically and statistically significant) because they underscore the relevance of using the distress thermometer and problem list (thus including the exploration of physical ailments) instead of other approaches to identify needs for psycho-oncological support (e.g., case finding).

Another limitation pertains to using the database of just one treatment center in Switzerland. This may influence the generalizability of findings. However, we were able to include a large set of data covering all cancer entities and types of treatment and a broad cohort of patients. Similarly, the DT and PL may not be particularly fine grained instruments to detect distress for research purposes, yet these instruments are the most commonly used in clinical practice internationally [38].

Finally, overfitting is a general problem in ML. We minimized this risk using different methods (see above). Still, it is important that our findings are validated in further research.

## 5. Conclusions

To the best of our knowledge, our study is the first one aiming to characterize patients declining psycho-oncological support for distress that focuses on a large set of clinically recorded data beyond the level of distress using a sophisticated methodological approach. From a clinical perspective, nurses’ cancer distress screening via the DT and PL is functional and valuable. Nurses should be enabled to do so in terms of training and time available for screening in clinical practice. For older patients, low-threshold support (e.g., non-specific or visiting services) might promote their readiness to accept support for cancer-related distress. From a research perspective, the results provide evidence for the usefulness of current internationally used screening procedures using the distress thermometer and problem list. Future research should explore how the training of nurses in resolving misconceptions about psycho-oncological support in patients could be optimized and what resources nurses need for proper screening.

## Figures and Tables

**Table 1:** Study group characteristics.

	Female	Male	Total
**Age**, Mean (SD)	60 (14.61)	62 (13.14)	62 (13.74)
**Age Group 18–64**, N, (%)	844/2150 (39%)	1306/2150 (61%)	2150/4064 (53%)
**Age Group 65–70**, N (%)	533/1618 (33%)	1085/1618 (67%)	1618/4064 (40%)
**Age Group 80+**, N (%)	117/296 (40%)	179/296 (60%)	296/4064 (7%)
**Distress Score**, Mean (SD)	4.81 (2.7)	3.9 (2.66)	4.29 (2.71)
**Physical Score**, Mean (SD)	4.6 (3.84)	3.44 (3.3)	3.86 (3.41)
**Wish**, N (%)	261/528 (49%)	267/528 (51%)	528/4064 (13%)
**No Wish**, N (%)	1233/3526 (35%)	2303/3536 (65%)	3536/4064 (87%)
	**Wish**	**No Wish**	**Total**
**Age**, Mean (SD)	58 (2.58)	62 (13.5)	62 (13.74)
**Age Group 18–64**, N, (%)	322/2150 (15%)	1828/2150 (85%)	2150/4064 (53%)
**Age Group 65–70**, N (%)	186/1618/11%)	1432/1618 (89%)	1618/4064 (40%)
**Age Group 80+**, N (%)	20/296 (7%)	276/296 (93%)	296/4064 (7%)
**Distress Score**, Mean (SD)	6.01 (2.58)	4.03 (2.62)	4.29 (2.71)
**Physical Score**, Mean (SD)	5.92 (3.88)	3.56 (3.23)	3.86 (3.41)
**Female**, N (%)	261/1494 (17%)	1233/1494 (83%)	1494/4064 (37%)
**Male**, N (%)	267/2570 (10%)	2303/2570 (90%)	2570/4064 (63%)

Note. Distress Score = distress score on the distress thermometer (0 = no distress, 10 = maximum distress); SD = standard deviation; No Wish = patients who decline psycho-oncological support; Wish = patients who accept psycho-oncological support; Physical Score = number of physical problems mentioned in the PL.

**Table 2:** Machine learning models and performance in nested CV.

Statistical Algorithm	Balanced Accuracy (%)	AUC	Sensitivity (%)	Specificity (%)	PPV (%)	NPV (%)
Logistic Regression	69.1	0.75	73.5	64.9	93.7	25.6
Tree	65.3	0.66	69.9	66.6	93.7	23.8
Random Forest	65.3	0.75	84.4	45.1	91.7	30.2
Gradient Boosting	69.3	0.76	83.5	64.3	93.7	38.1
KNN	58.8	0.65	79.5	37.6	90.1	20.4
SVM	69.5	0.75	74.4	64.9	93.8	26.3
Naive Bayes	69.2	0.79	70.7	67.1	93.9	24.4

Note. AUC = receiver operating characteristic balanced area under the curve; PPV = positive predictive value (for the outcome variable); NPV = negative predictive value (for the outcome variable); KNN = k nearest neighbors, SVM = support vector machines.

**Table 3:** Final Naïve Bayes model performance measures on testing set.

Performance Measures	%	95% Confidence Interval
Balanced Accuracy	68.5	[64.4, 71.9]
AUC	0.76	[0.72, 0.80]
Sensitivity	88.9	[88.8, 89]
Specificity	47.2	[46.9, 47.4]
PPV	90.8	[90.7, 90.8]
NPV	42.2	[42, 42.4]

Note. AUC = receiver operating characteristic balanced area under the curve; PPV = positive predictive value (for the outcome variable); NPV = negative predictive value (for the outcome variable).

**Table 4:** Absolute and relative distribution of indicative variables.

Variable Description	No Wish	Wish
Age, Mean	62.2	57.9
Distress Score, Mean	4.03	6.09
Physical Score, Mean	3.56	5.92
CCI, Mean	2.03	2.7
Fear, N (%)	1325/3461 (38.3)	353/518 (68.1)
Worries, N (%)	1383/3455 (40)	359/517 (69.4)
Sadness, N (%)	779/3437 (22.7)	308/516 (59.7)

Note. DT = Distress Thermometer; PL = Problem List; CCI = Charlson Comorbidity Index; Fear/Worries/Sadness = Patients who check-marked Fear/Worries/Sadness in the PL.

## Data Availability

The data that support the findings of this study are available on request from the corresponding author. The data are not publicly available due to privacy or ethical restrictions.

## Appendix A

**Table A1:** List of Variables Used.

Variable	Description
WISH_PO	Wish for psychological referral (yes/no)
SEX	Gender (woman/men)
Versich = A	Insurance basic (yes/no)
Versich = HP	Insurance medium (yes/no)
Versich = P	Insurance premium (yes/no)
Alter bei Screening	Age at time of screening
Alter 18–64	Age between 18 and 64 (yes/no)
Alter 65–79	Age between 65 and 79 (yes/no)
Alter > 80	Age 80 or higher (yes/no)
Wert DT	Distress Score (on the DT (1–10))
DT ≥ 5	score of 5 or higher (yes/no)
Probl/Prakt	Any item of the category “practical” on the problem list (yes/no)
Probl/Prakt/Wohnen	Item “living” on the problem list (yes/no)
Probl/Prakt/Versich	Item “insurance” on the problem list (yes/no)
Probl/Prakt/ArbeitSchule	Item “work/school” on the problem list (yes/no)
Probl/Prakt/Transp	Item “transport” on the problem list (yes/no)
Probl/Prakt/KindBetr	Item “child care” on the problem list (yes/no)
Probl/Fam	Any item of the category “family” on the problem list (yes/no)
Probl/Fam/Partner	Item “partner” on the problem list (yes/no)
Probl/Fam/Kind	Item “child” on the problem list (yes/no)
Probl/Emotion	Any item of the category “emotion” on the problem list (yes/no)
Probl/Emotion/Sorgen	Item “worries” on the problem list (yes/no)
Probl/Emotion/Angst	Item “fear” on the problem list (yes/no)
Probl/Emotion/Traurig	Item “sadness” on the problem list (yes/no)
Probl/Emotion/Depress	Item “depression” on the problem list (yes/no)
Probl/Emotion/Nervos	Item “nervous” on the problem list (yes/no)
SUMMEEMO	Total amount of items of the category “emotion”
Probl/Spirituel	Any item of the category “spiritual” on the problem list (yes/no)
Probl/Spirituel/Gott	Item “god” on the problem list (yes/no)
Probl/Spirituel/VerlGlaub	Item “loss of faith” on the problem list (yes/no)
Probl/Physis	Any item of the category “physical” on the problem list (yes/no)
Probl/Physis/Nausea	Item “nausea” on the problem list (yes/no)
Probl/Physis/Schmerz	Item “pain” on the problem list (yes/no)
Probl/Physis/Erschoepfung	Item “exhaustion” on the problem list (yes/no)
Probl/Physis/Schlaf	Item “sleep” on the problem list (yes/no)
Probl/Physis/Mobilit	Item “mobility” on the problem list (yes/no)
Probl/Physis/WaschAnkl	Item “washing/getting dressed” on the problem list (/no)
Probl/Physis/Atmung	Item “breathing” on the problem list (yes/no)
Probl/Physis/AErsBild	Item “appearance” on the problem list (yes/no)
Probl/Physis/EntzuMundb	Item “mouth sores” on the problem list (yes/no)
Probl/Physis/Essen	Item “eating” on the problem list (yes/no)
Probl/Physis/Verdaustor	Item “Indigestion” on the problem list (yes/no)
Probl/Physis/Diarhoe	Item “diarrhea” on the problem list (yes/no)
Probl/Physis/Obstipation	Item “Constipation” on the problem list
Probl/Physis/AendUrini	Item “changes in urination” on the problem list (yes/no)
Probl/Physis/Fieber	Item “fever” on the problem list (yes/no)
Probl/Physis/TrkJukHaut	Item “itchy and dry skin” on the problem list (yes/no)
Probl/Physis/KribbelnHF	Item “tiringly in hands/feet” on the problem list (yes/no)
Probl/Physis/Aufgedun	Item “feeling swollen” on the problem list (yes/no)
Probl/Physis/Sex	Item “sexuality” on the problem list (yes/no)
Probl/Physis/TrkVersNase	Item “Nose dry/congested” on the problem list (yes/no)
SUMMEPHYS	Physical Score: Total amount of items of the category “physical”
Zvilstand Paartnerschaft	relationship status “in a relationship” (yes/no)
Zvilistand getrennt/verwitwet/geschieden	relationship status “separated”, “widowed” or “divorced” (yes/no)
Zivilstand Ledig	relationship status “unwed” (yes/no)
Sprache DE	Language German (yes/no)
Sprach nicht DE	Language not German (yes/no)
KON_EV	protestant confession (yes/no)
KON_KATH	catholic confession (yes/no)
KON_OHNE	no confession (yes/no)
KON_AND	other confession (yes/no)
Schweizer/in?	Nationality Swiss (yes/no)
Europa	Nationality European country (yes/no)
Low/middle	From a low or middle income country (yes/no)
PFJ 2011	Year of diagnosis 2011 (yes/no)
PFJ 2012	Year of diagnosis 2012 (yes/no)
PFJ 2013	Year of diagnosis 2013 (yes/no)
PFJ 2014	Year of diagnosis 2014 (yes/no)
PFJ 2015	Year of diagnosis 2015 (yes/no)
PFJ 2016	Year of diagnosis 2016 (yes/no)
PFJ 2017	Year of diagnosis 2017 (yes/no)
PFJ 2018	Year of diagnosis 2018 (yes/no)
PFJ 2019	Year of diagnosis 2019 (yes/no)
Brust	breast cancer (yes/no)
Darm	gastrointestinal cancer (yes/no)
Endo	endocrinological cancer (yes/no)
GenitalFrau	gynecological cancer (yes/no)
Hämato/Leukämie/Lymphom	hematological cancer incl. Leukemia and lymphoma (yes/no)
Harnblase	bladder cancer (yes/no)
Haut	skin cancer (yes/no)
Hoden/Penis	Penis or testicle cancer (yes/no)
Kopf/Hals	head and neck cancer (yes/no)
Lunge	lung cancer (yes/no)
Neuro	neurological cancers (yes/no)
Pankreas	pancreatic cancer (yes/no)
Prostata	prostatic cancer (yes/no)
Leber	Liver cancer (yes/no)
Akademisch	profession requiring academic degree (yes/no)
Unbekannt	Unknown employment status (yes/no)
Rentner	retired (yes/no)
Sonst	other profession (yes/no)
IV	disability pension (yes/no)
Arbeitslos	unemployed (yes/no)
Antidepressiva	antidepressant prescribed (yes/no)
Antiepileptika	mood stabilizer/antiepileptic medication prescribed (yes/no)
Antipsychotika	antipsychotic medication prescribed (yes/no)
Benzodiazepine	benzodiazepine prescribed (yes/no)
Glukokortikoide systemisch	steroids prescribed (yes/no)
Nichtopioidanalgetika	non-opioid analgesics prescribed (yes/no)
Opioidanalgetika	opioids prescribed (yes/no)
Psychopharmaka	any psychopharmacology prescribed (yes/no)
Schmerzmittel	Any painkiller prescribed (yes/no)
CCI	Charlson comorbidity index (1–5)
F0	Mental disorders due to known physiological conditions diagnosed (yes/no)
F1	Mental and behavioral disorders due to psychoactive substance use diagnosed (yes/no)
F2	Schizophrenia, schizotypal, delusional, and other non-mood psychotic disorders diagnosed (yes/no)
F3	Mood [affective] disorders diagnosed (yes/no)
F4	Anxiety, dissociative, stress-related, somatoform and other nonpsychotic mental disorders diagnosed (yes/no)
F5	Behavioral syndromes associated with physiological disturbances and physical factors diagnosed (yes/no)
F6	Disorders of adult personality and behavior diagnosed (yes/no)
F7	Intellectual disabilities diagnosed (yes/no)
F8	Pervasive and specific developmental disorders diagnosed (yes/no)
F9	behavioral and emotional disorders with onset usually occurring in childhood and adolescence and unspecific disorders diagnosed (yes/no)
Fdiagnose	Any mental disorder diagnosed (yes/no)

**Table A2:** Default Hyperparameters for Model Building during Nested Cross Validation.

Algorithm	Hyperparameters
Logistic Regression	-
Tree	minisplit = 20; cp = 0.01; maxcomplete = 4; maxsurrogate = 5; usesurrogate = 2; surrogatestyle = 0; maxdepth = 30;xval = 10
Random Forest	Ntree = 500; replace = true; nodesize = 1; importance = false; localImp = false;
Gradient Boosting	distribution = Bernoulli; n.tress = 100; cvfolds = 0; interaction.depth = 1; n.minobsinnode = 10; shrinkage = 0.1; bag.fraction = 0.5; train.fraction = 1
KNN	integer = 7; numeric = 2; logical = true
SVM	cost = 1; nu = 0.5; kernel = radial; degree = 3; cachesize = 40; tolerance = 0.001; shrinking = true
Naive Bayes	laplace = 0

**Table A3:** Detailed Performance Results with Confidence Intervals (CI) for Model Building during Nested Cross Validation.

ML Algorithm	Balanced Accuracy (95% CI)	AUC (95% CI)	Sensitivity (95% CI)	Specificity (95% CI)	PPV (95% CI)	NPV (95% CI)
Logistic Regression	69.1 (62.7–74.4)	0.75 (0.68–0.82)	73.5 (73.3–73.6)	64.9 (64.5–65.2)	93.7 (93.6–93.8)	25.6 (25.4–25.8)
Tree	65.3 (62.4–73.9)	0.66 (0.59–0.73)	69.9 (69.8–70.1)	66.6 (66.2–66.9)	93.7 (93.6–93.8)	23.8 (23.6–23.9)
Random Forest	65.3 (59.7–71.5)	0.75 (0.68–0.82)	84.4 (85.3–85.5)	45.1 (44.8–45.5)	91.7 (91.6–91.8)	30.2 (29.9–30.5)
Gradient Boosting	69.3 (63–74.7)	0.76 (0.69–0.83)	83.5 (83.4–83.6)	64.3 (63.9–64.6)	93.7 (93.6–93.7)	38.1 (37.9–38.4)
KNN	58.8 (53–64.8)	0.65 (0.58–0.72)	79.5 (79.4–79.6)	37.6 (37.3–38)	90.1 (90–90.2)	20.4 (20.2–20.6)
SVM	69.5 (63.2–74.8)	0.75 (0.68–0.82)	74.4 (74.1–74.6)	64.9 (64.5–65.2)	93.8 (93.7–93.9)	26.3 (26.1–26.5)
Naive Bayes	69.2 (62.7–74.2)	0.79 (0.72–85.5)	70.7 (70.6–70.8)	67.1 (66.8–67.5)	93.9 (93.8–93.9)	24.4 (24.2–24.5)

## References

[1] Fallowfield L., Ratcliffe D., Jenkins V., Saul J. (2001). Psychiatric morbidity and its recognition by doctors in patients with cancer. Br. J. Cancer.

[2] Zabora J., BrintzenhofeSzoc K., Curbow B., Hooker C., Piantadosi S. (2001). The prevalence of psychological distress by cancer site. Psychooncology.

[3] Wang G.L., Cheng C.T., Feng A.C., Hsu S.H., Hou Y.C., Chiu C.Y. (2017). Prevalence, risk factors, and the desire for help of distressed newly diagnosed cancer patients: A large-sample study. Palliat. Support. Care.

[4] Sharpe M., Strong V., Allen K., Rush R., Postma K., Tulloh A., Maguire P., House A., Ramirez A., Cull A. (2004). Major depression in outpatients attending a regional cancer centre: Screening and unmet treatment needs. Br. J. Cancer.

[5] Ownby K.K. (2019). Use of the Distress Thermometer in Clinical Practice. J. Adv. Pract. Oncol..

[6] Hollingworth W., Metcalfe C., Mancero S., Harris S., Campbell R., Biddle L., McKell-Redwood D., Brennan J. (2013). Are needs assessments cost effective in reducing distress among patients with cancer? A randomized controlled trial using the Distress Thermometer and Problem List. J. Clin. Oncol..

[7] Thombs B.D., Coyne J.C., Cuijpers P., de Jonge P., Gilbody S., Ioannidis J.P., Johnson B.T., Patten S.B., Turner E.H., Ziegelstein R.C. (2012). Rethinking recommendations for screening for depression in primary care. CMAJ.

[8] Mitchell A.J. (2013). Screening for cancer-related distress: When is implementation successful and when is it unsuccessful?. Acta Oncol..

[9] van Scheppingen C., Schroevers M.J., Smink A., van der Linden Y.M., Mul V.E., Langendijk J.A., Coyne J.C., Sanderman R. (2011). Does screening for distress efficiently uncover meetable unmet needs in cancer patients?. Psychooncology.

[10] Tuinman M.A., Gazendam-Donofrio S.M., Hoekstra-Weebers J.E. (2008). Screening and referral for psychosocial distress in oncologic practice: Use of the Distress Thermometer. Cancer.

[11] Clover K., Kelly P., Rogers K., Britton B., Carter G.L. (2013). Predictors of desire for help in oncology outpatients reporting pain or distress. Psychooncology.

[12] Baker-Glenn E.A., Park B., Granger L., Symonds P., Mitchell A.J. (2011). Desire for psychological support in cancer patients with depression or distress: Validation of a simple help question. Psychooncology.

[13] Clover K.A., Mitchell A.J., Britton B., Carter G. (2015). Why do oncology outpatients who report emotional distress decline help?. Psychooncology.

[14] Holland J.C., Andersen B., Breitbart W.S., Compas B., Dudley M.M., Fleishman S., Fulcher C.D., Greenberg D.B., Greiner C.B., Handzo G.F. (2010). Distress management. J. Natl. Compr. Cancer Netw..

[15] Stanton A.L., Wiley J.F., Krull J.L., Crespi C.M., Weihs K.L. (2018). Cancer-related coping processes as predictors of depressive symptoms, trajectories, and episodes. J. Consult. Clin. Psychol..

[16] Charlson M.E., Pompei P., Ales K.L., MacKenzie C.R. (1987). A new method of classifying prognostic comorbidity in longitudinal studies: Development and validation. J. Chronic. Dis..

[17] The World Bank (2020). Current Classification by Income.

[18] Dwyer D.B., Falkai P., Koutsouleris N. (2018). Machine Learning Approaches for Clinical Psychology and Psychiatry. Annu. Rev. Clin. Psychol..

[19] Browne M.W. (2000). Cross-Validation Methods. J. Math. Psychol..

[20] Bischl B., Lang M., Kotthoff L., Schiffner J., Richter J., Studerus E., Casalicchio G., Jones Z.M. (2016). mlr: Machine Learning in R. J. Mach. Learn. Res..

[21] Ishwaran H., Kogalur U.B. (2020). Package ‘randomForestSRC’: Fast Unified Random Forests for Survival, Regression, and Classification (RF-SRC). 2.9.3. http://www.est.colpos.mx/R-mirror/web/packages/randomForestSRC/randomForestSRC.pdf.

[22] Wei Q., Dunbrack R.L. (2013). The role of balanced training and testing data sets for binary classifiers in bioinformatics. PLoS ONE.

[23] James G., Witten D., Hastie T., Tibshirani R. (2013). An Introduction to Statistical Learning.

[24] Campbell G. (1994). Advances in statistical methodology for the evaluation of diagnostic and laboratory tests. Stat. Med..

[25] Fitzgerald P., Lo C., Li M., Gagliese L., Zimmermann C., Rodin G. (2015). The Relationship between Depression and Physical Symptom Burden in Advanced Cancer. BMJ Support Palliat Care.

[26] Petrova D., Redondo-Sánchez D., Rodríguez-Barranco M., Romero Ruiz A., Catena A., Garcia-Retamero R., Sánchez M.J. (2021). Physical comorbidities as a marker for high risk of psychological distress in cancer patients. Psychooncology.

[27] VanHoose L., Black L.L., Doty K., Sabata D., Twumasi-Ankrah P., Taylor S., Johnson R. (2015). An analysis of the distress thermometer problem list and distress in patients with cancer. Support. Care Cancer.

[28] Borson S., Korpak A., Carbajal-Madrid P., Likar D., Brown G.A., Batra R. (2019). Reducing Barriers to Mental Health Care: Bringing Evidence-Based Psychotherapy Home. J. Am. Geriatr. Soc..

[29] Holland J.C. (2002). History of psycho-oncology: Overcoming attitudinal and conceptual barriers. Psychosom. Med..

[30] Wei W., Sambamoorthi U., Olfson M., Walkup J.T., Crystal S. (2005). Use of psychotherapy for depression in older adults. Am. J. Psychiatry.

[31] Stark A., Kaduszkiewicz H., Stein J., Maier W., Heser K., Weyerer S., Werle J., Wiese B., Mamone S., König H.H. (2018). A qualitative study on older primary care patients’ perspectives on depression and its treatments—Potential barriers to and opportunities for managing depression. BMC Fam. Pract..

[32] Gühne U., Luppa M., Stein J., Wiese B., Weyerer S., Maier W., König H.-H., Riedel-Heller S.G. (2016). “Die vergessenen Patienten”—Barrieren und Chancen einer optimierten Behandlung depressiver Erkrankungen im Alter. Psychiatr. Prax..

[33] Kacel E.L., Pereira D.B., Estores I.M. (2019). Advancing supportive oncology care via collaboration between psycho-oncology and integrative medicine. Support. Care Cancer.

[34] Gühne U., Luppa M., König H.H., Riedel-Heller S.G. (2014). Collaborative and home based treatment for older adults with depression: A review of the literature. Nervenarzt.

[35] Merckaert I., Libert Y., Messin S., Milani M., Slachmuylder J.L., Razavi D. (2010). Cancer patients’ desire for psychological support: Prevalence and implications for screening patients’ psychological needs. Psychooncology.

[36] Nekolaichuk C.L., Cumming C., Turner J., Yushchyshyn A., Sela R. (2011). Referral patterns and psychosocial distress in cancer patients accessing a psycho-oncology counseling service. Psychooncology.

[37] Rajesh A., Stefanek M. (2022). Controversies in Psycho-Oncology.

[38] Donovan K.A., Grassi L., McGinty H.L., Jacobsen P.B. (2014). Validation of the distress thermometer worldwide: State of the science. Psychooncology.

